# Morphological, Histochemical, and Proteomic Analysis of the Effects of Fluoride and Amoxicillin, with Calcium and Vitamin D Supplementation, on Dental Enamel Formation

**DOI:** 10.1007/s00223-026-01548-0

**Published:** 2026-05-25

**Authors:** I. M. Porto, A. A. S. da Silva, J. V.A. dos Santos, B. M. Silva, E. B. Giacomin, G. S. da Fonseca, R. F. Gerlach

**Affiliations:** 1 Department of Morphology and Pediatric Clinic, São Paulo State University (FOAr-UNESP), Araraquara, São Paulo, Brazil; 2 Department of Morphology and Genetics, Federal University of São Paulo, São Paulo, Brazil; 3 Department of Basic and Oral Biology, Faculty of Dentistry of Ribeirão Preto, University of Sao Paulo (DBBO/FORP/USP), Ribeirao Preto, São Paulo, Brazil

**Keywords:** Enamel, Ameloblasts, Fluoride, Amoxicillin, Calcium

## Abstract

Ameloblasts are specialized epithelial cells responsible for enamel formation, a process that requires tightly coordinated matrix secretion, proteolytic turnover, and hydroxyapatite crystal growth. Although genetically regulated, amelogenesis is susceptible to environmental modulation by agents such as fluoride and amoxicillin. Calcium and vitamin D supplementation have been proposed as potential protective factors against fluoride-induced enamel alterations. This study investigated whether combined exposure to fluoride and amoxicillin impairs ameloblast viability and enamel formation, and whether vitamin D and calcium supplementation can mitigate these effects. Using a rat incisor model, enamel structure, ameloblast apoptosis, and matrix protein dynamics were evaluated through ultrastructural analysis, histology, immunohistochemistry, TUNEL assay, and proteomic profiling. Fluoride exposure, particularly when combined with amoxicillin, resulted in disorganization of enamel prisms and increased apoptotic activity in ameloblasts, as evidenced by caspase-3 and TUNEL labeling, especially during the early maturation stage. Immunohistochemistry revealed reduced KLK4 levels within ameloblasts, whereas proteomic analysis demonstrated increased KLK4 abundance in the enamel matrix, suggesting altered intracellular trafficking and impaired proteolytic turnover. Additionally, the abundance of AMBN, AMTN, and ODAM, proteins essential for enamel crystal nucleation and maturation, was reduced in treated groups. Vitamin D and calcium supplementation neither significantly restored enamel organization nor normalized matrix protein alterations under the experimental conditions tested. Overall, these results indicate that fluoride exposure, particularly when combined with amoxicillin administration, disrupts ameloblast homeostasis, matrix proteolysis, and enamel prism organization, resulting in structurally compromised enamel, with no beneficial effects from vitamin D and calcium supplementation.

**Supplementary Information:**

The online version contains supplementary material available at 10.1007/s00223-026-01548-0.

## Introduction

Dental enamel is the most highly mineralized tissue in the body, comprising more than 95% mineral by weight at the completion of its mineralization process [1]. For complete enamel maturation to occur, the organic matrix must be almost entirely removed from the enamel matrix [2, 3]. During the mineralization stage, the protease responsible for cleaving enamel proteins is kallikrein-4 (KLK4), a serine protease secreted by ameloblasts [4–5]. Although amelogenesis is genetically regulated [6], it can be affected by external factors such as fluoride and amoxicillin [7, 8].

Excess fluoride exposure during enamel mineralization disrupts enamel formation and leads to dental fluorosis [9]. Fluorotic enamel exhibits reduced hardness, increased organic matrix retention, and decreased mineral content [10, 11]. Protein retention is particularly evident at the enamel surface of fluorotic rat incisors [12]. Notably, mass spectrometry analyses of human fluorotic enamel reveal no differences in peptide sequence patterns compared with controls, suggesting that proteolytic processing is not markedly altered [13]. Rather, peptide persistence within the enamel matrix may impair crystal growth and maturation, consistent with earlier reports showing that fluoride does not significantly modify enamel protein content or protease activity [10, 14, 15].

Enamel maturation appears particularly vulnerable to fluoride toxicity. Cyclical extracellular acidification during mineral influx promotes the formation of hydrogen fluoride, which diffuses into ameloblasts and dissociates in the neutral cytosol, leading to intracellular fluoride accumulation [16]. This triggers endoplasmic reticulum stress, activation of the unfolded protein response, impaired protein synthesis, and ameloblast apoptosis, providing a cellular basis for defective mineralization.

Antibiotic exposure during prenatal and early postnatal periods has also been implicated in enamel disturbances. Frequent amoxicillin administration has been associated with enamel alterations in animal models, especially following prenatal exposure [17, 18]. Experimental studies report altered ameloblast morphology, reduced enamel matrix secretion, and hypomineralization characterized by decreased calcium and phosphate content and reduced KLK4 expression [18–20]. Epidemiological data further suggest increased fluorosis prevalence in children exposed concomitantly to fluoride and frequent amoxicillin use [21, 22], although experimental findings on their interaction remain inconsistent [19, 23].

Mineral availability may influence fluorosis severity. Calcium and vitamin D supplementation during enamel formation has been associated with attenuation of fluorosis [24, 25], whereas calcium deficiency induces reversible enamel hypomineralization [26].

Thus, although fluoride and amoxicillin have been independently linked to enamel alterations, it remains unclear whether their combined exposure exerts additive or synergistic effects on enamel mineralization and ameloblast function, and whether mineral supplementation may mitigate these changes. The present study was designed to address these questions.

## Methods

### Animals and Experimental Design

The experimental protocol was approved by the Ethics Committee of the School of Dentistry of Araraquara (process no. 01/2023). A total of 80 male Holtzman rats (Rattus norvegicus albinus), aged 2–3 months and weighing approximately 200 g at baseline and approximately 500 g at the end of the 60-day treatment period, were used in this study.

Animals were treated for 60 days with fluoride, amoxicillin, and calcium plus vitamin D, administered either individually or in combination. Rats were randomly assigned to eight experimental groups (*n* = 10 per group):

(I) control, (II) fluoride, (III) fluoride + amoxicillin, (IV) amoxicillin, (V) fluoride + vitamin D + calcium, (VI) amoxicillin + vitamin D + calcium, (VII) vitamin D + calcium, and (VIII) fluoride + amoxicillin + vitamin D + calcium.

At the end of the treatment period, animals were euthanized. Urine samples were collected for fluoride quantification; hemimandibles were harvested for morphological analyses (light microscopy, transmission and scanning electron microscopy, and immunohistochemistry); and upper and lower incisors were collected for phosphate quantification and proteomic analysis.

#### Description of Experimental Groups

Control: animals received water and food *ad libitum* for 60 days.

Fluoride: animals received 45 mg fluoride/L (100 mg sodium fluoride/L) in drinking water for 60 days, with food provided *ad libitum*.

Vitamin D + Calcium (VitD + Ca): animals received 2.0 g calcium carbonate/150 g chow and 7000 IU/week vitamin D, administered intragastrically, for 60 days. Water and food were provided *ad libitum*.

Amoxicillin (Amx): animals received amoxicillin (500 mg/kg body weight) as an oral suspension, administered intragastrically every 24 h for 60 days, following Souza et al. (2021). Water and food were provided *ad libitum*.

Fluoride + Amoxicillin (F + Amx): animals received 45 mg fluoride/L in drinking water and amoxicillin (500 mg/kg body weight) administered intragastrically every 24 h for 60 days.

Fluoride + Vitamin D + Calcium (F + VitD + Ca): animals received 45 mg fluoride/L in drinking water, 2.0 g calcium carbonate/150 g chow, and 7000 IU/week vitamin D for 60 days, with food and water *ad libitum*.

Amoxicillin + Vitamin D + Calcium (Amx + VitD + Ca): animals received amoxicillin (500 mg/kg/day), 2.0 g calcium carbonate/150 g chow, and 7000 IU/week vitamin D for 60 days. Water and food were provided *ad libitum*.

Fluoride + Amoxicillin + Vitamin D + Calcium (F + Amx + VitD + Ca): animals received fluoride, amoxicillin, calcium, and vitamin D according to the protocols described above, with water and food provided *ad libitum* for 60 days.

The fluoride dose (45 ppm) was selected based on previous studies demonstrating its effectiveness in inducing enamel mineralization defects under controlled experimental conditions [11]. The amoxicillin dose (500 mg/kg/day) was chosen according to prior investigations reporting enamel malformation in treated rats [17]. The vitamin D and calcium supplementation protocol was based on previous evidence suggesting that these nutrients may reduce the severity of fluoride-induced enamel alterations [25]. Throughout the experimental period, daily food and water intake per animal were recorded, and body weight was monitored every 15 days.

#### Urinary Fluoride Quantification

Urinary fluoride concentration was determined to assess fluoride excretion. On the day of euthanasia, urine was directly aspirated from the bladder using a 1-mL syringe fitted with a fine-gauge needle. Samples were stored at − 20 °C until analysis.

Prior to measurement, urine samples were mixed with TISAB II (pH 5.0) at a 1:1 ratio. Fluoride concentration was determined using a fluoride-selective ion electrode (Orion Star A214 Analyzer) calibrated with standard solutions ranging from 0.125 to 32 µg F/mL. Results are expressed as mg F/L. Differences between groups were analyzed by One-way ANOVA. The significance level was set as 0.05 (*p* < 0.05).

#### Macroscopic Analysis of the Lower Incisors

The labial surface of the lower incisors was photographed to record alterations caused by fluoride and amoxicillin in the brownish coloration of the teeth.

#### Quantification of Phosphorus Released Following Acid Etching

Phosphorus quantification was used to determine whether calcium and vitamin D supplementation enhance the resistance of superficial enamel to acid challenge. Five rat incisors per group were used. Briefly, the labial surface of the incisal third of the lower incisors was immersed for 20 s in 500 µL of 1.8% nitric acid.

Phosphorus concentration was determined using a colorimetric method, as described by Costa de Almeida et al. [27]. Measurements were performed in triplicate at 660 nm using a spectrophotometer (DR 2500, Hach, Loveland, USA). Calibration curves were generated using phosphorus standards at 1, 2, 4, and 8 mg/mL. Differences between groups were analyzed by One-way ANOVA. The significance level was set as 0.05 (*p* < 0.05).

#### Scanning Electron Microscopy (SEM)

Five lower incisors per group were ground under water cooling to a thickness of 500 μm, as measured with a digital caliper. Specimens were etched with 37% phosphoric acid gel for 30 s, sonicated for 10 min, fixed in 4% paraformaldehyde, dehydrated through a graded acetone series, mounted on aluminum stubs, carbon-coated, and examined by scanning electron microscopy.

#### Light Microscopy

Light microscopy was used to evaluate enamel matrix structure and ameloblast morphology. Five hemimandibles per group were dissected and fixed in 4% buffered paraformaldehyde (pH 7.2) for 48 h. Specimens were decalcified in 7% EDTA (pH 7.2), dehydrated through graded ethanol, cleared in xylene, and embedded in paraffin. Section  (5 μm) were obtained and stained with hematoxylin and eosin (H&E).

## TUNEL Assay

Deparaffinized sections mounted on silanized slides were processed using the ApopTag Peroxidase In Situ Apoptosis Detection Kit (Merck, USA), according to the manufacturer’s instructions. Sections were pretreated with proteinase K (20 µg/mL; Sigma-Aldrich) for 15 min at room temperature, followed by incubation with 3% hydrogen peroxide to quench endogenous peroxidase activity. Terminal deoxynucleotidyl transferase (TdT) labeling was performed at 37 °C for 1 h. The reaction was stopped, and sections were incubated with anti-digoxigenin–peroxidase for 30 min. Immunoreactivity was visualized using 3,3′-diaminobenzidine (DAB; Sigma-Aldrich), followed by counterstaining with Carazzi’s hematoxylin.

### Immunohistochemistry

Immunohistochemistry for kallikrein-4 and cleaved caspase-3 was performed as follows: deparaffinized sections were subjected to antigen retrieval in sodium citrate buffer (pH 6.0) using a pressure cooker at 90 °C for 30 min. Endogenous peroxidase activity was quenched with 3% hydrogen peroxide. Sections were then incubated overnight at 4 °C with anti-cleaved caspase-3 (Cell Signaling Technology, USA) or anti-kallikrein-4 (Abcam, ab231048, USA) diluted 1:200. Immunodetection was performed using the Vectastain Elite ABC Universal Plus Kit (Vector Laboratories), with ImmPACT DAB used as the chromogen. Sections were counterstained with Carazzi’s hematoxylin. For each slide containing two serial sections, one section served as a positive control, while the other served as a negative control in which the primary antibody was omitted and replaced with PBS.

### KLK4 Immunolabeled Area Quantification

The measurement of the KLK 4 immunolabelled areas was performed in images acquired with a Leica DFC 550 camera attached to a Leica BM4000 B LED microscope, and a Leica Application Suite (LAS 4.3) software. The KLK 4 immunolabelled area was measured across two non-consecutive sections per animal. The analysis was performed within a standardized total ameloblast epithelial area (post secretory ameloblast region), and the immunopositive area per µm² of epithelium was calculated for KLK 4 marker. To ensure consistency, parameters for threshold, color range, hue, and saturation were kept constant for the marker.

*Ameloblast layer height* due to the difficulty in identifying the end of the secretory stage and the onset of enamel mineralization, the height of ameloblasts in the region where KLK4 quantification was performed, was measured to ensure that, across groups, the ameloblasts were at the same stage of amelogenesis (post-secretory stage). The ameloblast layer height was measured in two non-consecutive sections per animal, in each Sect.  3 pictures of the post secretory ameloblast region were taken and the epithelial height in µm was measured three times in each picture, totaling 30 measurements per animal.

### Statistical Analysis

Statistical analyses were performed using GraphPad Prism 10.2.3 software (GraphPad Software, CA, USA). To examine whether the samples were normally distributed, the normality test (Shapiro–Wilk test) was applied. Differences between groups were analyzed by One-way ANOVA. The significance level was set as 0.05 (*p* < 0.05).

#### Liquid Chromatography Mass Spectrometry (LC–MS/MS) Analysis

Five upper incisors per group were used for protein extraction. The labial enamel surface was immersed for 5 min in 500 µL of 10% HCl containing protease inhibitors (N-ethylmaleimide, phenanthroline, and phenylmethylsulfonyl fluoride; 2 mM each). Protein extracts were desalted using ZipTip C18 tips (Merck Millipore) and eluted with 50% acetonitrile/0.1% trifluoroacetic acid (TFA).

Peptides were analyzed by LC–MS/MS using an Orbitrap Exploris 240 mass spectrometer (Thermo Fisher Scientific, USA) coupled to an Evosep One liquid chromatography system via a nanoelectrospray ion source, operated in data-independent acquisition (DIA) mode. Peptides were loaded onto EV2001 C18 Evotips and separated on an IonOpticks Aurora Elite™ XT C18 UHPLC column (15 cm × 75 μm, 1.7 μm particle size) using the Evosep Whisper Zoom 20SPD method (20 samples/day).

The nanoelectrospray voltage was set to 1.7 kV, and the ion source temperature was maintained at 275 °C. Full MS scans were acquired at a resolution of 120,000 over an m/z range of 350–1400, with an automatic gain control (AGC) target of 300% and a maximum injection time of 45 ms. MS/MS spectra were acquired using 34 DIA windows, each spanning 20 m/z units (361–1033 m/z), at a resolution of 15,000, with an AGC target of 1000% and a normalized collision energy of 27%.

#### Proteomic Data Processing

Data-independent acquisition (DIA) raw files were processed using Spectronaut v19 (Biognosys, Zurich, Switzerland) with the directDIA workflow and default settings, against the Rattus norvegicus UniProt database (22,403 entries; downloaded September 2025). Peptide identification was performed using a no-enzyme (unspecific) digestion setting, allowing peptide lengths of 7–52 amino acids, up to two missed cleavages, and up to five variable modifications. Methionine oxidation and protein N-terminal acetylation were specified as variable modifications. Identification thresholds were set to precursor Q-value < 0.01, precursor posterior error probability (PEP) < 0.2, protein (experiment) Q-value < 0.01, protein (run) Q-value < 0.05, and protein PEP < 0.75. Protein-level quantitative values (PG.Log2Quantity) were exported from the Experiment Analysis report (PG_Quantity sheet) at the protein group (PG.ProteinGroups) aggregation level. Only protein groups with non-missing quantitative values were retained for downstream analyses. Exported intensities were already log₂-transformed, and no additional global transformation was applied. MS2-based quantities (PG.MS2Quantity) were not used for the primary differential abundance analysis. For each experiment, runs were classified according to the Run label defined in Spectronaut and used to generate three independent pairwise comparisons: fluoride vs. control, amoxicillin vs. control, and fluoride + amoxicillin vs. control. For each contrast, PG.Log2Quantity values corresponding to control and treated samples were extracted from the PG_Quantity sheet and grouped by experimental condition.

#### Per-Protein Summarization and Differential Abundance Analysis

All statistical analyses were performed in R (version 2025.09.2). For each pairwise comparison, a working dataset (pg_stats) was generated from the PG_Quantity sheet and included protein group identifiers (PG.ProteinGroups), gene symbols (PG.Genes), log_2_-transformed quantitative values for individual runs (PG.Log2Quantity), and derived summary statistics. For each protein group and comparison, the mean log_2_ intensity was calculated separately for control and treated samples as the mean across all runs within each condition (rowMeans, na.rm = TRUE). The log_2_ fold change (log_2_FC) was defined as:

$$ {\mathrm{log}}_{{2}} {\text{FC }} = {\text{ mean}}\_{\text{treated }} - {\text{ mean}}\_{\mathrm{control}} $$.

with positive values indicating higher protein abundance in the treated group and negative values indicating higher abundance in controls.

Differential abundance was assessed using a two-sided unpaired Student’s t-test applied to log_2_ intensities for each protein group. Resulting p values were calculated using the base R t.test function. Proteins with insufficient variability or entirely missing values across conditions were assigned NA and excluded from statistical inference. Correction for multiple testing within each comparison was performed using the Benjamini–Hochberg procedure, yielding the false discovery rate (FDR). The primary criterion for differential abundance was FDR < 0.05. Given the modest sample size and the limited number of proteins meeting this stringent threshold, additional exploratory criteria were applied, as described below.

##### Volcano and Venny Plot Visualization

For data visualization and hypothesis generation, volcano plots were constructed for each pairwise comparison using ggplot2. For each protein group, log_2_ fold change (logFC) was plotted on the x-axis, and either − log_10_(FDR) or − log_10_(*p* value) was plotted on the y-axis, as appropriate. Vertical dashed lines were drawn at |log_2_FC| = 0.58, corresponding to an approximate 1.5-fold change, and horizontal dashed lines were placed at FDR = 0.05 or nominal *p* = 0.05.

Because none or only a limited number of proteins met the stringent FDR < 0.05 criterion, exploratory candidate sets were defined using more permissive thresholds. First, proteins with |log₂FC| > 0.58 ( ≈ ≥ 1.5-fold change), irrespective of FDR, were used to describe overall patterns of protein up- and downregulation across treatments. Second, proteins with |log_2_FC| > 0.58 and nominal *p* < 0.05 (unadjusted) were highlighted in volcano plots (red, increased abundance in treated groups; blue, decreased abundance) and exported; their corresponding FDR values were reported but not used as strict cutoffs.

To visualize treatment-associated changes in key proteins, heatmaps were generated based on log_2_ fold-change (log_2_FC) values derived from the differential proteomic analysis (treated vs. control). For each comparison (fluoride vs. control, amoxicillin vs. control, and fluoride + amoxicillin vs. control), protein-level log_2_FC values were calculated from the log_2_-transformed Spectronaut intensities as described above. Additionally, a Venny diagram [28] was constructed with the proteins with downregulated abundance and the proteins related to enamel formation were highlighted.

The union of all selected genes across the three comparisons was used to construct a gene × contrast matrix (rows = proteins; columns = fluoride, amoxicillin, fluoride + amoxicillin).

All R scripts used for data processing and visualization are available from the corresponding author upon reasonable request.

## Results

### Average Daily Water and Food Intake

The addition of sodium fluoride to drinking water and calcium carbonate to the diet did not impair palatability, as daily water and food intake were comparable to those observed in the control group (data not shown). Based on fluoride concentration in drinking water and average daily intake, estimated fluoride ingestion was approximately 100-fold higher in fluoride-treated groups (2.10–2.42 mg/animal/day) compared with non-fluoride–treated groups (0.02–0.03 mg/animal/day).

#### Urinary Fluoride

Urinary fluoride concentration in rats treated with fluoride was markedly higher, as expected (mean 8.33 mgF/L ± 3.99 in fluoride-treated groups versus 0.76 mgF/L ± 0.35 in the other groups). This indicates that, in animals receiving sodium fluoride salts added to the drinking water, the amount of circulating fluoride in the organism was substantially higher—on the order of tenfold—compared with groups in which fluoride was not added to the water. The table containing urinary fluoride concentrations is presented in Supplementary Table 1.

#### Disruption of Brownish Pigmentation 

**Fig. 1:**
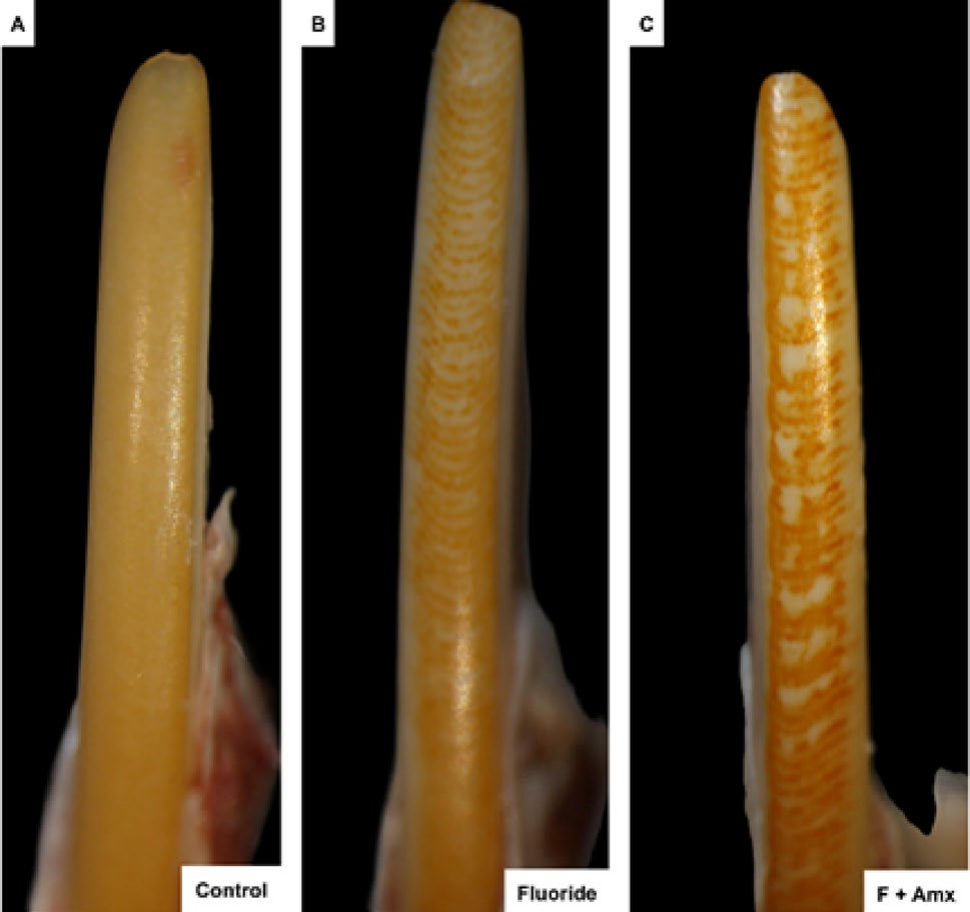
Photographs of the lower incisors.**A** Control group. **B** Fluoride group. **C** Fluoride plus amoxicillin group (F + Amx). Enamel striations are observed in the fluoride-treated group (**B**) and are more pronounced in the group treated concomitantly with amoxicillin (**C**). Original magnification: 10×

The lower incisors of the fluoride-treated groups exhibited defects in the brownish pigmentation, showing a pattern of alternating light and dark striations. The width of these striations was increased in the teeth of animals treated with fluoride + amoxicillin. The remaining groups (including those treated with amoxicillin alone) did not show any alterations (Fig. 1).

#### Vitamin D and Calcium Supplementation did not Enhance Mineralization of the Enamel Surface or its Internal Structure

Phosphorus concentration following acid etching of the labial surface of the lower incisor was calculated. The mean values (mg/ml P ± SD) were as follows: Control (4.81 ± 2.39), VitD + Ca (3.66 ± 1.72), Amx (3.96 ± 1.96), Amx + VitD + Ca (2.39 ± 1.53), Fluoride (5.46 ± 2.42), F + VitD + Ca (2.44 ± 1.19), F + Amx (4.50 ± 2.02), and F + Amx + VitD + Ca (4.42 ± 2.09). This finding indicates that calcium and vitamin D supplementation did not significantly alter the resistance of superficial enamel to acid challenge.

**Fig. 2:**
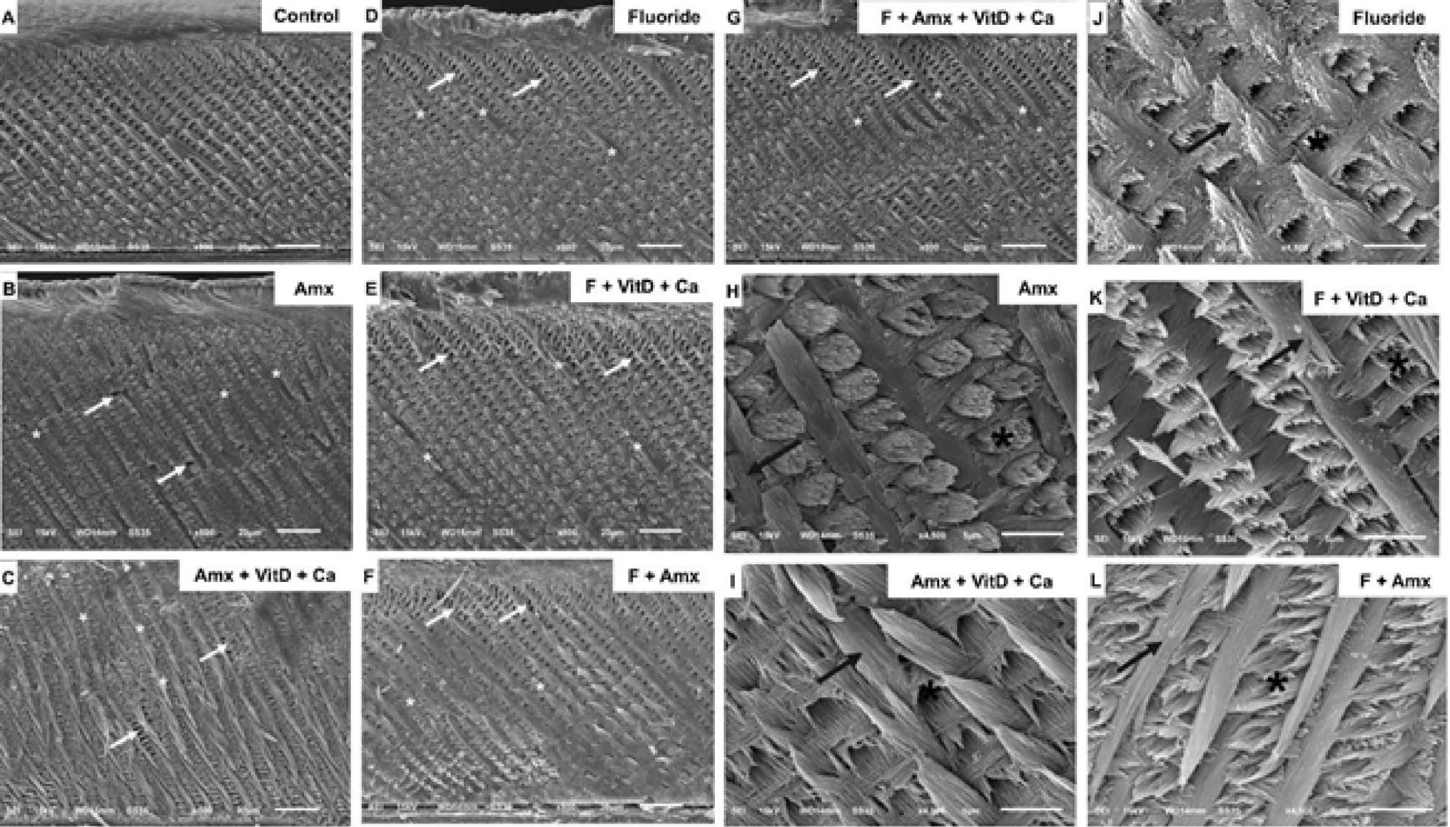
Scanning electron microscopy (SEM). (**A**–**G**) Experimental groups exhibit thinner enamel prisms compared with the control group (white asterisks), while the interprismatic enamel shows increased width (white arrows). Magnification: 800×. (**H**–**L**) Representative higher-magnification images (4,500×) demonstrate structural disruption of both prismatic enamel (black arrows) and aprismatic enamel (white asterisks). Structural disorganization was more pronounced in the fluoride-treated groups

Scanning electron microscopy revealed disorganization of hydroxyapatite prisms in the groups treated with fluoride and/or amoxicillin, administered either individually or in combination. In these groups, the prismatic enamel appeared thinner, resulting in a wider interprismatic enamel band and exhibiting focal voids within the enamel matrix. These ultrastructural alterations persisted in animals receiving calcium and vitamin D supplementation, indicating that supplementation did not prevent deeper prism disorganization associated with fluoride and amoxicillin exposure (Fig. 2). Notably, prism disorganization was more pronounced in the fluoride-treated groups.

#### Fluoride and Amoxicillin Induce Ameloblasts Death

**Fig. 3:**
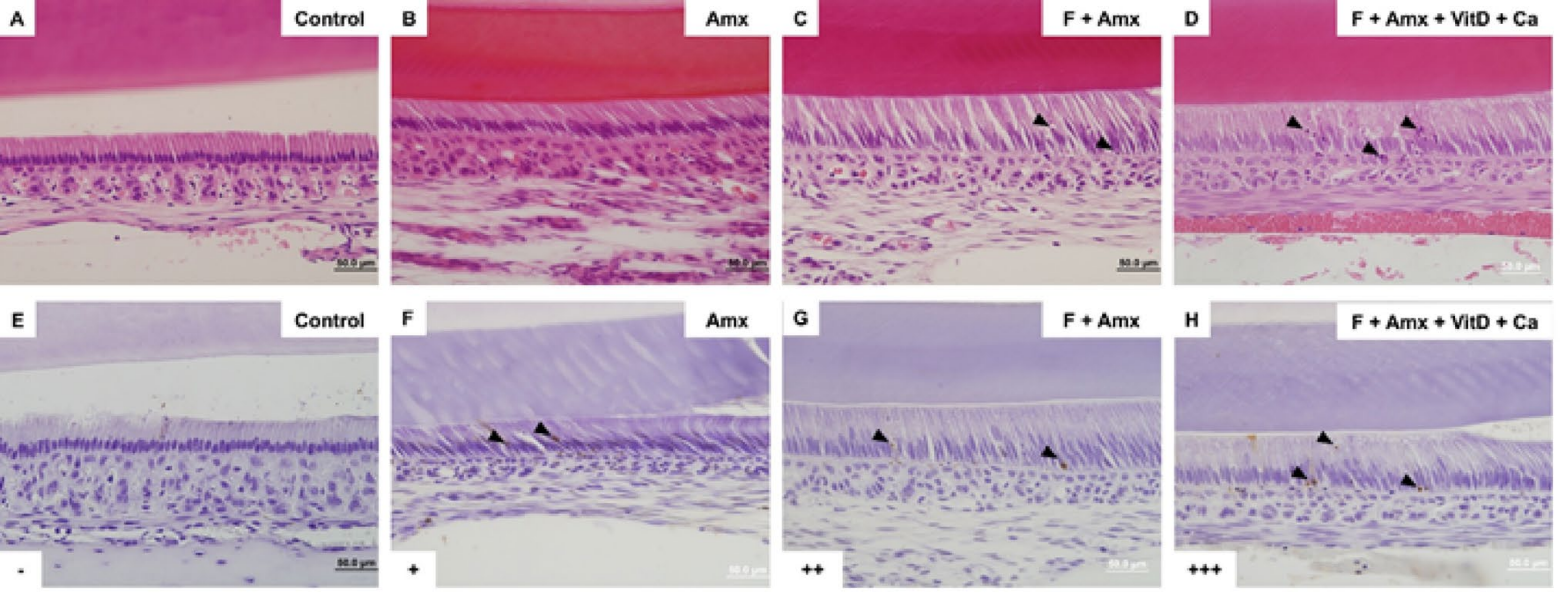
(**A**–**D**) Hematoxylin and eosin staining. (**E**–**H**) TUNEL assay. In the H&E staining, the amoxicillin, fluoride plus amoxicillin, and fluoride plus amoxicillin plus vitamin D and calcium groups showed the presence of apoptotic bodies (arrowheads). Apoptosis was confirmed by positive TUNEL assay (arrowheads). Original magnification: 400×. Qualitative assessment of apoptotic bodies. Scoring criteria: (-) absence of apoptotic bodies; (+) few apoptotic bodies; (++) moderate number of apoptotic bodies; (+++) large number of apoptotic bodies

H&E-stained sections revealed apoptotic bodies in ameloblasts and within the enamel organ epithelium, particularly near the proximal region, in the amoxicillin, fluoride + amoxicillin, and fluoride + amoxicillin + vitamin D + calcium groups. These alterations were most evident during the early post secretory stage of amelogenesis (Figs. 3a–d), in the enamel region located approximately 1.5 mm from the cervical loop. The TUNEL assay confirmed the presence of cell death, with DAB-positive nuclei and apoptotic bodies identified both within ameloblasts and in the adjacent enamel organ epithelium (Figs. 3f–h). The apoptotic process was more pronounced in the group that received concomitant administration of fluoride and amoxicillin (Fig. 3h).

**Fig. 4:**
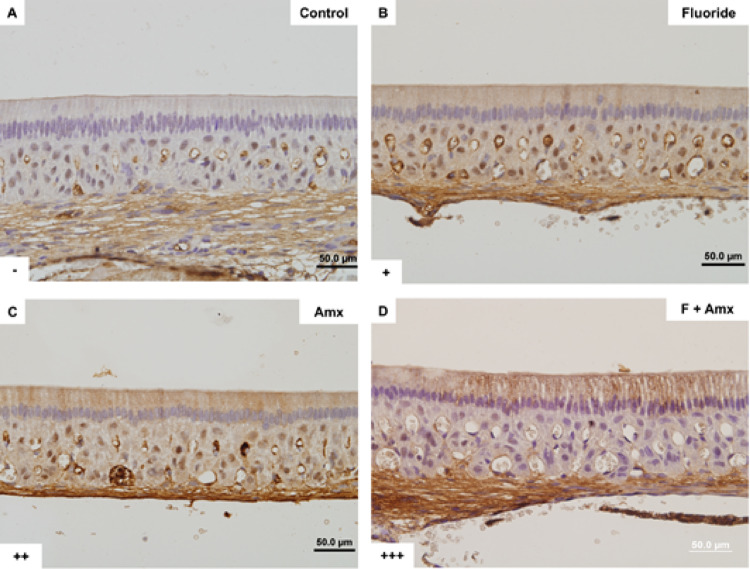
Immunohistochemistry for cleaved caspase-3. Positive caspase-3 immunoreactivity is indicated by brown staining in the cytoplasm of ameloblasts. The fluoride (**B**), amoxicillin (**C**), and fluoride plus amoxicillin (**D**) groups showed positive caspase-3 immunoreactivity (arrowheads), with stronger labeling particularly observed in the fluoride plus amoxicillin group. The control group (**A**) did not show positive caspase-3 staining. Original magnification: 400×. Qualitative analysis of caspase-3 immunostaining in dental enamel. Scoring criteria: (–) negative for caspase-3; (+) low caspase-3 positivity; (++) moderate caspase-3 positivity; (+++) high caspase-3 positivity

Immunohistochemical analysis for cleaved caspase-3 revealed positive immunolabeling in ameloblasts from the fluoride, amoxicillin, and fluoride + amoxicillin groups (Fig. 4b–d). The positive reaction to cleaved caspase-3 was more pronounced in the groups that received fluoride + amoxicillin (Fig. 4d). Calcium and vitamin D supplementation did not modify the differences observed among the experimental groups, nor did it promote significant changes when compared to the control group (data not shown). More intense caspase-3 immunolabeling was observed in ameloblasts located approximately 3 mm from the cervical loop, corresponding to a more mineralized enamel region, compared with areas in which apoptotic bodies were detected.

#### Fluoride Treatment Causes Disturbances in KLK4

**Fig. 5:**
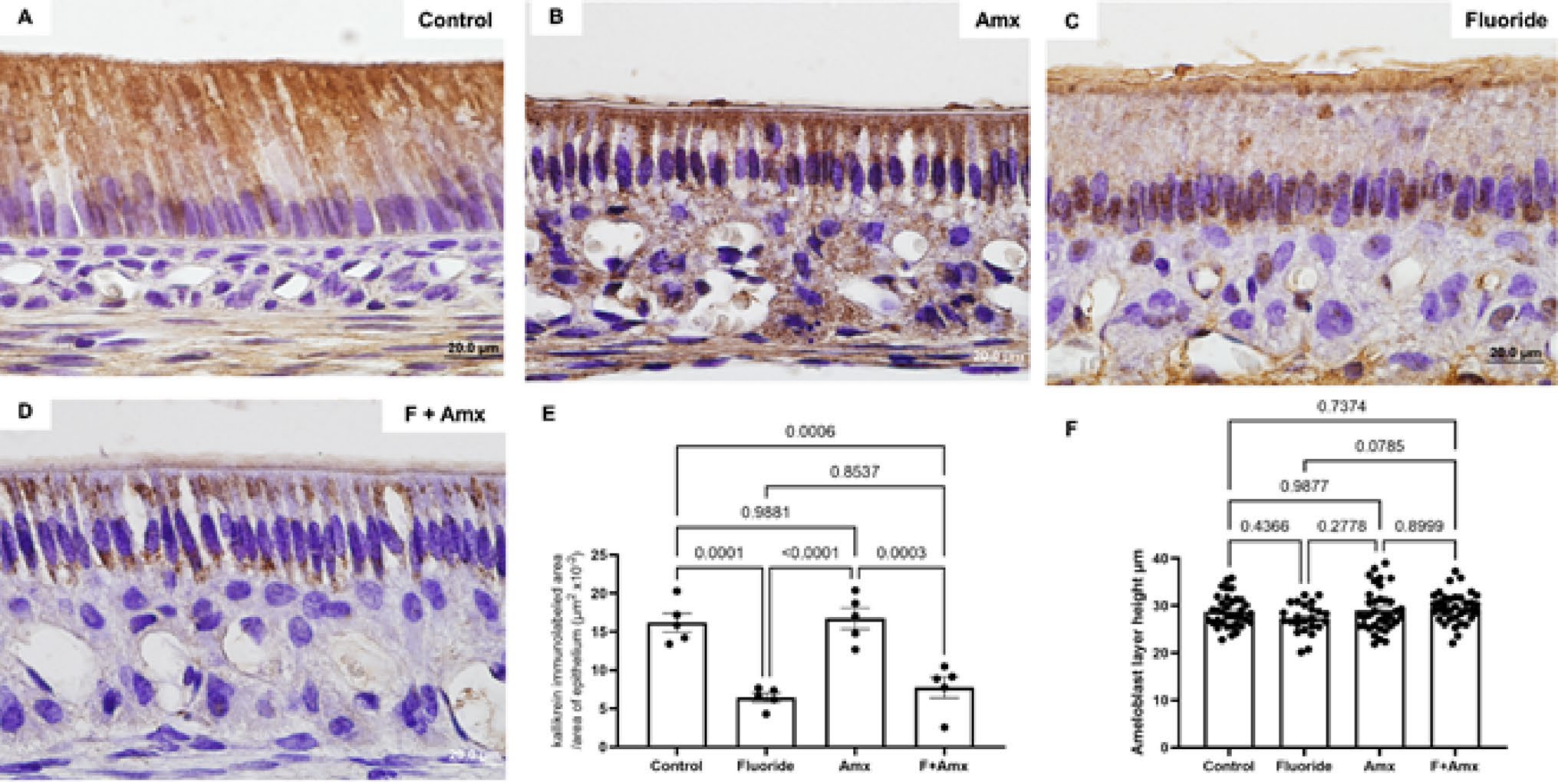
Immunohistochemistry for KLK4. The fluoride-treated groups (**C**–**D**) showed reduced KLK4 immunoreactivity compared with the control and amoxicillin groups (**A**–**B**). This difference was statistically significant (**E**) (*p* < 0.01). Measurement of ameloblast height (µm) in the region used for KLK4 immunolabeling showed no significant differences among groups (**F**), indicating that ameloblasts were in the same stage of amelogenesis (post-secretory stage).** A**–**D**: Original magnification: 1000×

Quantification of KLK4 in the ameloblast layer showed a statistically decreased immunolabeling of this protease within ameloblasts, indicating that fluoride interferes with the production of this protein by ameloblasts. Measurements of the ameloblast layer in which KLK4 quantification was performed showed no differences among groups, confirming that the analyses were carried out at the same stage of amelogenesis (post-secretory stage) (Fig. 5). Calcium and vitamin D supplementation did not modify the differences observed among the experimental groups, nor did it promote significant changes when compared to the control group.

#### Fluoride and Amoxicillin Treatment Alters Enamel Proteins Abundance

**Fig. 6:**
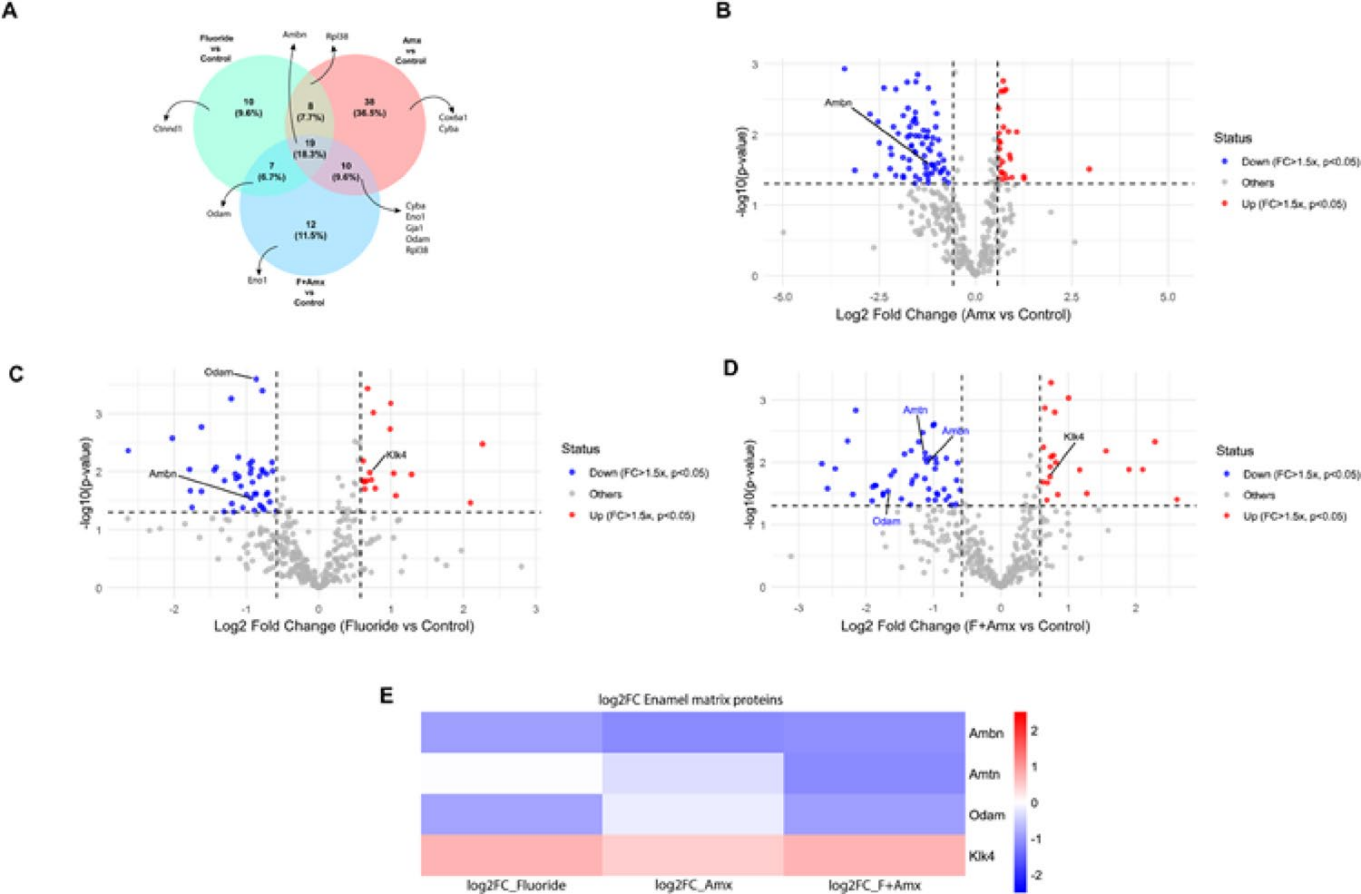
Proteomic analysis of enamel matrix proteins identified by LC–MS/MS. (**A**) Venn diagram and (**B**–**D**) volcano plot visualization of differentially expressed proteins relative to the control group. (**A**) Venn diagram of proteins with downregulated abundance in the amoxicillin (Amx), fluoride, and fluoride plus amoxicillin (F + Amx) groups. Proteins functionally associated with ameloblast biology and enamel matrix organization are highlighted. AMBN was the only consistently downregulated protein shared among groups. (**B**) Amx group exhibited reduced abundance of AMBN. (**C**) Fluoride group showed reduced abundance of AMBN and odontogenic ameloblast-associated protein (ODAM), together with increased abundance of kallikrein-4 (KLK4). (**D**) F + Amx group showed reduced abundance of AMBN, ODAM, and amelotin (AMTN), along with increased abundance of KLK4. (**E**) Heatmap summarizing log₂ fold-change in enamel matrix protein abundance in experimental groups relative to control. Significant changes were defined as fold change ≥ 1.5 and *p* < 0.05

Overall, the amoxicillin group exhibited the highest number of downregulated proteins. Focusing on proteins closely related to ameloblast metabolic activity or enamel matrix mineralization, both the amoxicillin and fluoride + amoxicillin groups showed the strongest downregulation profiles. Ameloblastin (AMBN) was consistently downregulated across all experimental groups compared to controls (Fig. 6a).

Comparative proteomic analysis between the fluoride-treated and control groups demonstrated that the enamel matrix of fluorotic animals exhibited increased abundance of KLK4, accompanied by reduced abundance of odontogenic ameloblast-associated protein (ODAM) and AMBN (fold change ≤ 1.5; *p* < 0.05) (Fig. 6b).

In the comparison between the amoxicillin-treated and control groups, the experimental group showed decreased abundance of AMBN (fold change ≤ 1.5; *p* < 0.05) (Fig. 6a).

Similarly, comparison of the fluoride + amoxicillin and control groups revealed downregulation of amelotin (AMTN) and AMBN, along with upregulation of KLK4 in the experimental group (fold change ≤ 1.5; *p* < 0.05) (Fig. 6c).

The heatmap comparing the three experimental groups with the control group revealed a greater inhibition of AMBN expression in the groups treated with amoxicillin. In contrast, ODAM showed reduced expression in the fluoride-treated groups. AMTN was downregulated exclusively in the fluoride plus amoxicillin (F + Amx) group. In addition, KLK4 abundance was increased in fluoride-treated groups, with a more pronounced upregulation in the combined fluoride + amoxicillin group (Fig. 6d). Calcium and vitamin D supplementation did not modify the differences observed among the experimental groups, nor did it promote significant changes when compared to the control group.

## Discussion

Proper dental enamel mineralization depends on the near-complete removal of the organic matrix and water, allowing mineral influx and hydroxyapatite crystal growth in thickness and width, a process strictly dependent on ameloblast functional integrity [29]. In the present study, although fluoride and/or amoxicillin exposure did not alter water or feed palatability, it induced significant structural and molecular alterations in enamel. Urinary fluoride concentrations confirmed the effective systemic exposure in animals receiving fluoride in the drinking water, indicating that the observed enamel alterations occurred under conditions of sustained circulating fluoride levels. A limitation of this study is the fact that fluoride was not measured in plasma, and plasma fluoride levels together with urinary fluoride values could give a more precise toxicokinetics picture. Nonetheless, in our view this does not compromise the main conclusions of the work.

Consistent with phosphorus-based quantification after acid etching, vitamin D and calcium supplementation did not improve superficial mineralization. Likewise, ultrastructural analysis did not reveal detectable changes in prismatic organization of enamel. These findings partially diverge from studies reporting a protective effect of calcium supplementation against fluoride-induced enamel damage [31, 32], suggesting that the modulatory effect of essential nutrients may depend on dose, exposure duration, or the specific experimental model employed. Further investigations are warranted to elucidate the underlying mechanisms, particularly those involving vitamin D receptor activation and the regulation of intracellular calcium transport and signaling in ameloblasts. Such investigations would be crucial to elucidate the molecular pathways through which these factors may interfere with enamel biomineralization.

In agreement with our structural findings, previous studies reported no significant differences in enamel prisms between fluorotic and control groups 31,32], whereas Bronckers et al. [33] demonstrated fluoride-induced alterations in crystal morphology at the ultrastructural level. This discrepancy in the literature supports the notion that fluoride may affect enamel quality through mechanisms that are not always detectable solely by conventional ultrastructural assessment.

Importantly, our histological and histochemical analyses provide insight into the mechanisms underlying these structural outcomes. An increased number of apoptotic bodies and higher TUNEL positivity were observed in ameloblasts, particularly during the transition from the secretory to the maturation stage. In contrast, greater caspase-3 immunolabeling was detected in a more advanced stage of enamel mineralization. Notably, both TUNEL and caspase-3 positive reactions were more pronounced in the fluoride + amoxicillin group, suggesting a synergistic disruption of ameloblast homeostasis. This finding aligns with previous reports indicating that fluoride induces ameloblast stress and apoptosis, thereby compromising enamel matrix processing. Sahlberg et al. [19] described ameloblast detachment and altered enamel secretion following exposure to amoxicillin alone or combined with fluoride. Although Souza et al. [23] and Kumazawa et al. [34] did not observe significant enamel alterations with amoxicillin administration, differences in dosing regimen, exposure duration, and developmental stage may account for these discrepancies. Moreover, epidemiological and experimental evidence linking amoxicillin to enamel defects [20] further supports the biological plausibility of our findings.

At the molecular level, our proteomic and immunohistochemical data strengthen the link between ameloblast dysfunction and defective matrix processing. Reduced KLK4 expression in ameloblasts from fluoride-treated groups is consistent with reports by Suzuki et al. [35], who associated fluoride exposure with decreased KLK4 transcription secondary to cellular stress. Similarly, Li et al. [36] showed that fluoride induces oxidative stress–mediated apoptosis in ameloblasts, which may indirectly affect protease regulation and matrix processing. Although Tye et al. [37] suggested that fluoride does not directly impair KLK4 secretion, our finding of increased KLK4 retention within the enamel matrix suggests that fluoride may alter not only protein production but also its processing, activation, or removal. This hypothesis agrees with Den Besten et al. [38], who described fluoride-induced alterations in proteinase activity and reduced smooth-ended ameloblasts responsible for efficient matrix resorption.

The consistent reduction in AMBN abundance in treated groups is particularly relevant. Given its proposed role as a nucleator and scaffold for hydroxyapatite crystal formation [39, 40], decreased AMBN suggests early disruption of cell–matrix interactions during the secretory phase. This molecular alteration may precede and contribute to the structural disorganization observed. Furthermore, reduced AMTN and ODAM levels—proteins associated with mineralization-stage regulation and crystal nucleation [41] —indicate that fluoride, especially when combined with amoxicillin, interferes not only with early matrix deposition but also with final enamel maturation and surface hardening.

The hypothesis that fluoride increases protein secretion while impairing matrix removal [42] provides a conceptual framework that integrates our findings. Fluoride-induced reduction of smooth-ended ameloblasts [38] and the characteristic subsurface protein retention in fluorotic enamel [43, 44] are consistent with our observation of altered KLK4 dynamics and reduced abundance of key enamel proteins. Together, these data suggest that fluoride disrupts the balance between matrix secretion and degradation, leading to incomplete protein removal and compromised enamel architecture.

In summary, our results integrate structural, cellular, and proteomic evidence shows that fluoride exposure, potentiated by concomitant amoxicillin administration, affects multiple, interconnected axes of amelogenesis. By correlating apoptosis, impaired KLK-4, altered matrix protein abundance, and enamel prism disorganization, our findings provide mechanistic support for the hypothesis that enamel defects arise from cumulative disturbances in ameloblast viability and matrix maturation rather than from isolated alterations in mineral deposition alone.

## Supplementary Information

Below is the link to the electronic supplementary material.

Supplementary Material 1
