# Supplementary figures and images for: Morphological, Histochemical, and Proteomic Analysis of the Effects of Fluoride and Amoxicillin, with Calcium and Vitamin D Supplementation, on Dental Enamel Formation
Source: Calcif Tissue Int. 2026 May 25;117(1):89. doi: 10.1007/s00223-026-01548-0 (PMC13201332; doi:10.1007/s00223-026-01548-0)

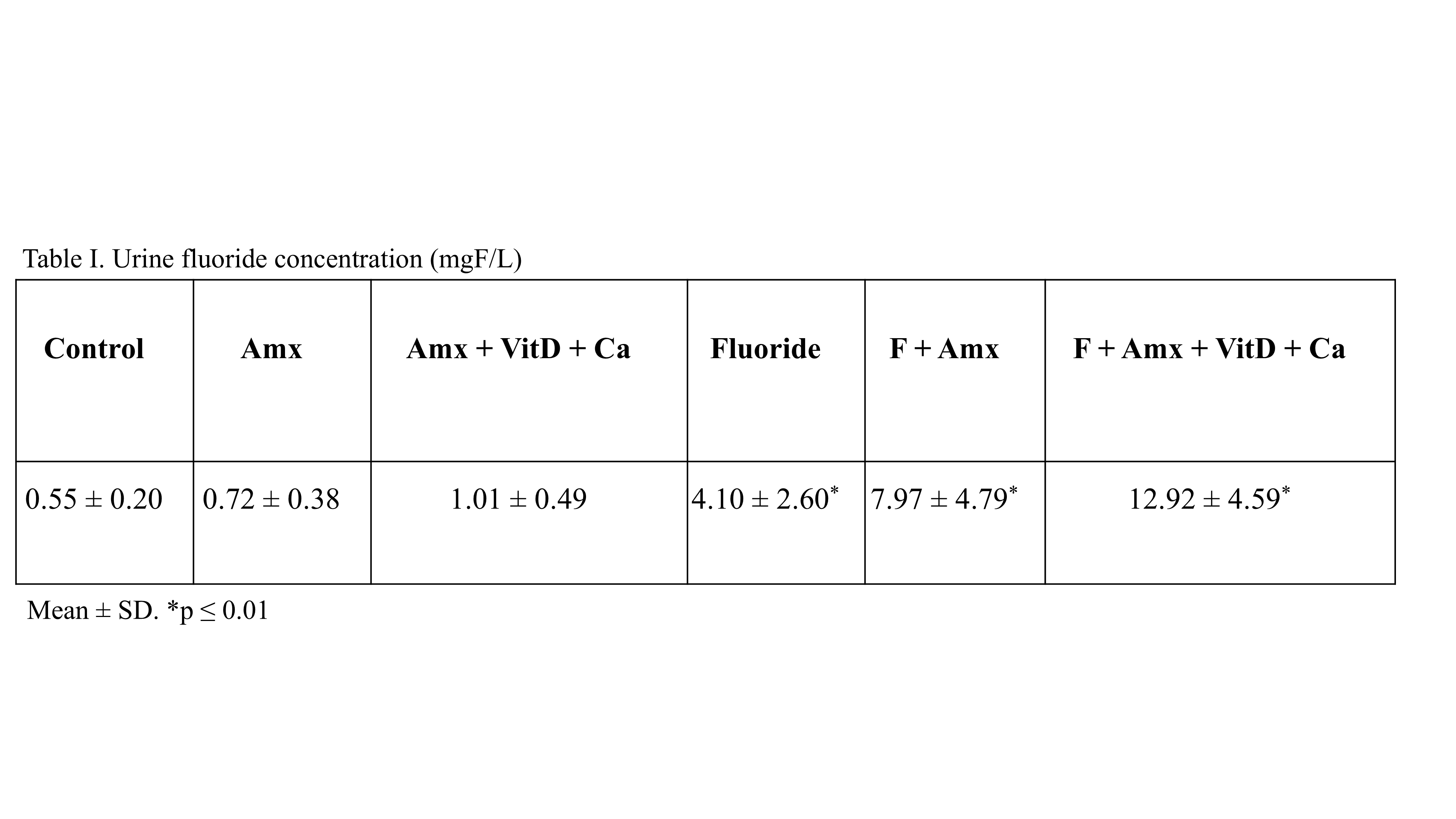

Supplement: Supplementary file 1 — Supplementary Material 1 [file 223_2026_1548_MOESM1_ESM.tif]
